# The Development and Consumer Acceptance of Functional Fruit-Herbal Beverages

**DOI:** 10.3390/foods9121819

**Published:** 2020-12-08

**Authors:** Sylwia Skąpska, Krystian Marszałek, Łukasz Woźniak, Justyna Szczepańska, Joanna Danielczuk, Katarzyna Zawada

**Affiliations:** 1 Department of Fruit and Vegetable Product Technology, Prof. Wacław Dąbrowski Institute of Agricultural and Food Biotechnology, 36 Rakowiecka Str., 02-532 Warsaw, Poland; krystian.marszalek@ibprs.pl (K.M.); lukasz.wozniak@ibprs.pl (Ł.W.); justyna.szczepanska@ibprs.pl (J.S.); joanna.danielczuk@ibprs.pl (J.D.); 2 Department of Physical Chemistry, Faculty of Pharmacy with the Laboratory Medicine Division, Medical University of Warsaw, 1 Banacha St., 02-091 Warsaw, Poland; kzawada@wum.edu.pl

**Keywords:** functional beverages, aronia, cranberry, sea buckthorn, acerola, rosehip, steviol glycosides, consumer tests

## Abstract

The development of functional beverages often requires a compromise between the palatability and high content of bio-active compounds. The purpose of this study was to elaborate on the fruit-herbal beverages with defined pro-health functions and evaluate their consumer acceptance. The beverages contained 80% of juices obtained from the fruits of aronia, rugosa rose, acerola, sea buckthorn, and cranberry. Each beverage was supplemented with different plant extracts which enhanced the designed functions of the beverage. The beverages were sweetened with sugar or with steviol glycosides, and were preserved by thermal pasteurization. The main groups of bio-active compounds and antioxidant capacity using ABTS, DPPH, and ORAC methods were analysed before and after pasteurization. The sensory acceptance was tested by 60 adult consumers who assessed the desirability of taste, odour, colour, and overall quality. Each beverage contained substantial amounts of polyphenols, including anthocyanins; rosehip-acerola and sea buckthorn beverages were also sources of vitamin C and carotenoids. All these components were stable under thermal treatment. Rosehip-acerola beverages had the highest antioxidant capacity, which was measured using all three methods exhibited. The highest level of consumer acceptance and willingness to purchase went to aronia beverages, while the sea buckthorn gained the lowest. There was no significant difference between the acceptance of beverages sweetened with sugar and stevia. Women and the 25- to 34-year-old consumer group rated the overall acceptability of the beverages slightly higher, although this was not reflected in their inclination to buy them. Attitude toward proper body mass and health had no influence on overall acceptance and willingness to complete the purchases. The main motivation for purchasing the functional beverages was their sensory acceptance, even if the consumers were informed of their potential health benefits.

## 1. Introduction

Among the many fruits of exceptional nutritional and pro-health values—the so-called superfruits—special attention should be paid to the aronia (*Aronia melanocarpa* Michx.), sea buckthorn (*Hippophaë rhamnoides* L.), rugosa rose (*Rosa rugosa* Thumb.), acerola (*Malpighia glabra* L.), and cranberry (*Oxycoccus palustris* Pers., *Vaccinium oxycoccus* L.).

All these fruits are rich sources of natural phenolic antioxidants; additionally, rosa rugosa, acerola, and sea buckthorn contain high amounts of vitamin C, while rosehip, acerola, and sea buckthorn have high amounts of carotenoids. The presence of all these compounds results in the remarkably high antioxidant potential of these fruits and the juices that are obtained from them, thus making them valuable foods in the prevention of diseases related to oxidative stress, such as cancer and cardiovascular diseases, and also in the strengthening of the immune system [1,2,3,4,5,6,7,8,9,10,11]. Moreover, each kind of fruit is characterized by specific, well-known, health-promoting properties that are widely used in traditional medicine. For example, the anti-inflammatory properties of galactolipids from rosehip are used in rheumatoid arthritis therapy and to support joint health [12]; aronia’s anthocyanins positively affect eyesight, and they also have an anticoagulant effect, strengthen the walls of blood vessels, and lower blood pressure [3,11]; the lipids present in sea buckthorn fruits contain bio-active tocopherols, carotenoids, and unsaturated fatty acids [13,14,15]; acerola is the richest source of vitamin C among all known fruits [16]; and cranberries are known for their ability to prevent and heal inflammation of the urinary tract [17].

All aforementioned fruits contain many valuable minerals, such as potassium, magnesium, and calcium, vitamins from the B, A, E, and K groups, and are rich in dietary fibre.

The nutritional and pro-health values of the juices obtained from the fruits listed above can be increased by their enrichment with plant extracts, especially herbal ones. There are many medicinal plant extracts of which could be applied to strengthen a selected prophylactic or therapy-supporting activity of a fruit beverage, such as ginger *(Zingiber officinale)* [18], nettle [19], or green tea [20]. They have been shown to possess, among other things, antioxidant, immunomodulatory, and antidiabetic properties, mainly due to their phenolic compounds [21].

There are many studies describing attempts to develop and characterize pro-healthy herbal-fruit beverages. Extracts of herbal plants were added to increase the health-promoting properties of beverages, but also to make their taste more attractive and increase the diversity of the offer of this type of product for consumers. For example, a beverage containing mosambi and lime juices with herbal extracts from tulsi, mint, and ginger was developed, containing several vitamins and minerals as well as other valuable components from herbs [22]. Other interesting ideas are lime juice supplemented with basil extract and ginger to increase the antioxidant potential and vitamin C content of the drink [23], and orange and celery juices with mint extract, sweetened with stevia, which are rich in carotene, iron, and calcium [24]. There have also been successful attempts at developing fruit and herbal drinks with whey, such as drinks containing whey, guava fruit juice, and extracts from basil, mint, ginger, aloe vera, and lemon grass [25] or pineapple juice and milk whey with *M. arvensis* extract [26]. Another innovative product of this kind is an orange beverage with nettle, containing probiotic bacteria *Lactobacillus rhamnosus* [19]. The research cited usually used locally grown fruits and herbs as a raw material.

When developing functional foods, it is important to not only provide an adequate amount of bioactive compounds, but also to ensure a favourable nutritional profile, which in the case of fruit products, such as limiting the sugar content. To ensure an acceptable quality of low-sugar products, part of or the whole amount of the added sugar can be replaced by a low-calorie sweetener. Recently, the most popular natural sweetener used for low-calorie beverages is steviol glycosides, obtained from the leaves of *Stevia rebaudiana,* of which rebaudioside A is the most sensory-accepted and has the best solubility in water; 200 times sweeter than sucrose, steviol glycosides do not provide any energy value and are stable up to 200 °C [27,28].

As has been proved by many researchers, the pro-health effect of novel and functional foods is not the prevailing motivation in consumer choices. Sensory quality is always the predominant factor determining acceptance and willingness to reach for the product [29,30,31]. Only a small segment of consumers is willing to compromise on taste for the health benefits of functional foods, and they are mainly older and of female gender [32].

Functional fruit products are usually prepared using raw materials with high polyphenol content, which are perceived as being bitter, tart, or astringent or with a high ascorbic acid content, and which is often associated with high acidity, reflected in its strong, sour taste. In the case of fruit-herbal drinks, herbs also usually have a specific bitter taste, which limits their use.

This study was aimed at determining consumer acceptance of newly developed fruit-herbal beverages with defined health-promoting functions, confirmed by an analysis of their composition and antioxidant capacities. The results were traced to find the correlations between age, gender, and attitude towards appropriate body mass and health and the willingness to consume such beverages.

## 2. Materials and Methods

### 2.1. Preparation of Beverages

All the beverages contained 80% of cloudy fruit juice, prepared by a Polish producer (Premium Rosa Sp. z o.o., Złotokłos, Poland) with the exception of acerola juice, which was imported from Brazil. The fresh fruits were washed, mashed in a mill (Rietz, Fort Worth, TX, USA), treated with commercial pectinolytic enzymes according to the producer’s instructions, and finally pressed on a belt press (Flottweg SE, Vilsbiburg, Germany). The cloudy juices were mixed with water (80:20 *v*/*v*), supplemented with extracts of cistus (*Cistus incanus*), green tea (*Camellia sinensis*), nettle leaves (*Urtica dioica)*, artichoke *(Cynara scolymus)*, Siberian ginseng *(Eleutherococcus senticosus),* ginger *(Zingiber officinale),* purple coneflower *(Echinacea purpurea),* aloe *(Aloe vera),* horsetail *(Equisetum arvense),* lingonberry *(Vaccinium vitis-idaea),* silver birch *(Betula pendula),* and chamomile *(Matricaria chamomilla),* which were kindly donated by GreenVit Sp. z o.o. and Vimax, Poland. The number of extracts was selected taking into account the dose recommended by the producer, as well as the sensory acceptance of the resulting product. The beverages were prepared in two versions: traditional—sweetened with sucrose and a dietary version of a 30% lower energy value, in which all added sugar was replaced by stevia extracts, containing 98% rebaudioside A (Lafar Co., Warsaw, Poland). All beverage components were mixed, heated to 80 °C, filtered, and filled into 250 mL dark glass bottles. Pasteurization was carried out in a batch pasteurizer at 85–87 °C for 10 min.

The samples before the pasteurization step (fresh samples) taken for analysis were frozen at −18 °C. The pasteurized samples were stored at room temperature and analysed for their physicochemical and sensory characteristics within one week after production.

### 2.2. Physicochemical Analysis of Beverages

The pH was measured using an HI 2210 pH meter (Hanna Instruments, Woonsocket, RI, USA).

The antioxidant capacity (AC) was determined using three different tests. The ORAC-fluorescein (ORAC-FL) assay was performed based on that proposed by Ou et al. [33]. The 30 μL volume of a sample diluted (300–1500 fold) with PBS (phosphate-buffered saline, pH 7.4). Standard Trolox solution or, in case of a blank, 30 μL of PBS buffer, were mixed with 180 μL of 112 nmol/L fluorescein solution in a well of a 96-well plate and kept for 15 min at 37 °C. Then, 100 μL of 100 mmol/L AAPH solution was added, and fluorescence was measured with a F-7000 Fluorescence Spectrophotometer (Hitachi, Tokyo, Japan) equipped with a Micro Plate Reader every 70 s for 90 min. The microplate was thermostated at 37 °C. The ABTS radical test was carried out based on the procedure of Re et al. [34]. The ABTS radical cation reagent was prepared by the reaction of ABTS with potassium persulfate, followed after 15 h with a dilution with ethanol to an absorbance of 0.700 (±0.01) at 734 nm. Then, 1500 μL of ABTS radical cation reagent was mixed with 15 μL of a sample (diluted 10–100 fold with ultrapure water) and after 6 min of reaction, the absorbance was measured with an EVOLUTION 60S spectrophotometer (Thermo Fisher Scientific). DPPH radical scavenging was measured as described by Sanna et al. [35]. The sample (10–100 μL, diluted 10-fold with ultrapure water) was mixed with the methanolic solution of DPPH (3.4 mmol/L), and after 40 min, EPR spectra were taken at ambient temperature (298 K) on a MiniScope MS200 EPR spectrometer (Magnettech). The intensity of registered EPR spectra was compared with the control sample (ultrapure water). All measurements were done in at least three repetitions. The results of all antioxidant capacity assays were expressed as Trolox equivalents (TE) in μmol TE per mililiter.

The total content of polyphenols (TCP) was measured spectrophotometrically according to Gao et al. [36]. The reaction mixtures were composed of 0.1 mL of sample, 2.0 mL of water, 0.2 mL of Folin–Ciocalteu reagent, and 1.0 mL of sodium carbonate solution (15%), and were incubated for 120 min at ambient temperature. The phenolic content was quantified by measurement of absorbance at the wavelength of 765 nm. The results were expressed as gallic acid equivalents.

The total anthocyanin content (TCA) was analysed using HPLC according to Oszmiański [37]. Briefly, 10 μL samples of juice were injected into Sunfire C18 5 μm, 4.6 × 250 mm column (Waters, Milford, MA, USA) and eluted for 28 min with a gradient of 4.5% formic acid and acetonitrile. The content of anthocyanins was determined using absorbance at wavelength at 520 nm; all concentrations were expressed as equivalents of cyanidin-3-glucoside.

### 2.3. Sensory Analysis and Consumer Aceptability Tests of Beverages

The preliminary sensory tests of beverage samples prepared in laboratory scale were performed by a trained nine-person sensory panel, using a nine-point hedonic scale for assessing taste, odour, colour, and overall quality [38]. The tests were aimed at perfecting the sensory quality of beverages and selecting the best recipe.

The consumer acceptance of pasteurized beverages prepared in industrial conditions was tested in a group of 60 adults. Participants were selected so as to represent five equal age groups with the same number of men and women in each group. The assessment took place in a sensory laboratory which met the requirements of ISO 8589:2007 [39]. The respondents were given survey questionnaires, with questions about their age, gender, and ensuring a proper body mass and good health (yes, rather yes, rather no, no). The questionnaires concerning product acceptance included an evaluation of the taste, odour, colour, and overall quality of the sample. In addition, there was a question about the willingness to buy these products (yes or no) if they appeared on the market. The beverages were served in 50 mL plastic transparent cups at room temperature. The participants received information on the composition of the product and the expected health effects.

The 11-point hedonic scale was used to assess the desirability of the product’s attributes (taste, odour, and colour) and overall quality, where 10 points meant the most desirable quality you can imagine; 5 points—indifferent; 0 points—the most undesirable you can imagine.

### 2.4. Statistical Analysis

The ANOVA with the post hoc Tukey test was used to evaluate the significance of the difference between the mean values of variables. The relationship between variables was assessed using the Pearson correlation coefficient. A chi-square test was applied to evaluate the influence of care for the proper body weight and health on the willingness to purchase beverages. A significant level α = 0.05 was applied to all the analyses. Statistica 13 (TIBCO Software Inc. 2017, Palo Alto, CA, USA) software was used for the statistics.

## 3. Results and Discussions

### 3.1. Recipes and Fuctions of Fruit-Herbal Beverages

The final recipes of four functional fruit-herbal beverages are shown in Table 1. All the beverages contained 80% of the juice. Three of them were prapared with a single type of fruit juice, and one was mixed (acerola and rosehip). The amount of added sugar in the traditional versions was 15–50 g/kg, depending on the sensory properties of the juice. The dietary version contained no added sugar, which was replaced by a sensory equivalent amount of steviol glycoside. The dietary versions had at least 30% less calories compared to their traditional counterpart, so they met the requirements of Regulation (EC) No. 1924/2006 [40] for products with reduced energy value. The beverages were positively assessed by a trained sensory panel according to taste, odour, colour, and overall quality, and each attribute was scored as over five points in a nine-point hedonic scale. 

The functions of the drinks resulted from the basic pro-health features of the fruit juices used. Herbal extracts were selected to support and enhance the functions of the designed beverage, according to their properties, described either in the European Pharmacopeia or ascribed to them in traditional medicine. Therefore, the main functions of the beverages were to be as follows: aronia—free radical scavenging, lowering of blood pressure, supporting the functioning of the liver and urinary tract, lowering the cholesterol level; rosehip-acerola—maintaining the normal functioning of the immune system, protecting cells against oxidative stress; cranberry—supporting the proper functioning of the urinary tract as well as the digestive system; sea buckthorn—maintaining healthy, good-looking skin, and strengthening of the hair and nails.

### 3.2. Physicochemical Properties of the Beverages

All the juices used for the production of beverages were moderately or strongly acidic, which was reflected in the pH values: 2.4 for cranberry, 2.7 for sea buckthorn, 3.4 for rosehip-acerola, and 3.5 for aronia beverage.

Each beverage was analysed for its content of the most important bio-active compounds derived from the fruit juice used (Table 2). We have analysed only the compounds that were present in reasonable amounts in each kind of fruit juice, which was checked in preliminary tests. As expected, the aronia beverages were rich in polyphenols, of which 21 to 31% were anthocyanins in fresh and pasteurized samples, respectively. Rosehip-acerola beverages showed a very high vitamin C content, they were also rich in carotenoids and polyphenols. Cranberry beverages contained substantial amounts of polyphenols and anthocyanis and sea buckthorn—vitamin C, carotenoids and polyphenols. All the studied compounds, with the exception of polyphenols, were slightly degraded during pasteurization.

Losses of vitamin C in rosehip-acerola beverages were small (11.7% and 7.9% in the traditional and dietetic versions, respectively), while in sea buckthorn, they were insignificant. Carotenoids were stable under heat treatment, and the maximum decrease in these compounds of 13.2% was observed in the dietetic sea buckthorn beverage. The changes in polyphenol content after pasteurization were insignificant in most cases, where the greatest decrease was in dietetic cranberry beverages, which amounted to 6.7%. The most thermo-labile compounds were anthocyanins. In cranberry beverages, losses of 49% and 38% were noted for the traditional and dietetic versions, respectively; aronia anthocyanins were more stable, with 37% and 20% decomposition for the traditional and dietetic versions, respectively.

Polyphenols, especially anthocyanins, are susceptible to degradation during processing. The greatest degree of degradation in these compounds during juice processing occurs due to the physical removal of the skins and seeds (pressing, clarification) and thermal processing (blanching, pasteurization) [41]. Many studies have reported a different degree of loss of polyphenols due to heating, depending on the severity and time of the treatment, and also the sensitivity of the different components to temperature. Far more significant changes occur when batch pasteurization lasting for a few to several dozen minutes is applied, compared to the UHT process, the time of which is counted in seconds [42,43]. The pasteurization process used in our study did not cause significant changes in the polyphenol content of the beverages, with one exception for the cranberry beverage sweetened with stevia, where a 15% decrease was observed (Table 2).

Anthocyanins were present in beverages based on aronia and cranberry juices (Table 2). The anthocyanins were the least stable of the bioactive constituents—in aronia beverages, their total content decreased by 38% and 20% for sugar and stevia-sweetened products, respectively, while in cranberry beverages, they decreased by 49% and 39%, respectively. Thermal degradation of anthocyanins was lower in stevia-sweetened samples, but the initial content of these pigments in two variants of aronia beverages differed, and was lower in diet beverage versions. This can be explained by the intermolecular co-pigmentation of these compounds and the protective sugar effect [44]. Wilkes and co-workers [45] reported a 60% degradation in the aronia anthocyanins during 10 min of pasteurization at 90 °C, while White et al. [46] reported an approx. 10% degradation after the same treatment for cranberries. Our previous study [47] also showed substantial anthocyanin degradation in aronia beverages subjected to thermal pasteurization.

The vitamin C content was analysed in two beverages that were developed to be rich sources of this compound (Table 2). The vitamin levels were 3.4–3.7 g/L in rosehip-acerola beverages and 1.1–1.2 g/L in the sea buckthorn ones. The Recommended Dietary Allowance (RDA) of vitamin C for adults is 75–90 mg per day, while the Tolerable Upper Intake Level (UL) is 2 g per day [48]. Therefore, the vitamin C levels in beverages ensure the administration of a sufficient daily intake of this vitamin in a 100 mL portion, while at the same time safeguarding against unintentional overdosing. Vitamin C proved to be stable under the processing conditions—none of the samples exceeded 5% loses during pasteurization. Additionally, Mercali et al. [49] reported an approx. 3% decrease in ascorbic acid in acerola pulps heated at 85 °C for 3 min. Relatively low losses of vitamin C can result from its high initial level. Samples with a lower initial vitamin C content are usually characterized by a higher degradation rate—Cvetković and Jokanović [50] reported an approx. 75% degradation in beverages containing vitamin C at levels of 100–150 mg/L. It is possible that the minor decrease in vitamin C levels in the developed beverages was also the result of a high content of antioxidants in the degradation of other compounds, which led to the depletion of oxygen in the beverages and where the low availability of oxygen strongly inhibits vitamin C degradation, even at elevated temperatures [51].

Rosehip, acerola, and sea buckthorn were the only sources of carotenoids among the used fruits. The content of these compounds was only slightly affected by the pasteurization process. Only one of the four tested variants, the sea buckthorn dietetic beverage, showed a statistically significant degradation in the carotenoids, which was still low, at approx. 13%. Other authors reported greater changes in the carotenoid content in pasteurized fruit beverages. Aaby et al. [52] reported a 54–100% recovery of carotenoids in pasteurized sea buckthorn puree, depending on the process parameters. Even a very short duration of heat treatment (10 s in 105 °C) used for orange juice pasteurization [53] resulted in more than 10% degradation in carotenoids. The minor changes in the carotenoids observed in our study could be the result of the presence of large amounts of other antioxidants preventing their degradation.

The presence of different groups of antioxidants was reflected in the antioxidant capacity (AC) of products, measured with three different assays. The highest values of AC expressed as Trolox equivalents in most of the samples were obtained using the ORAC method and the lowest using the ABTS assay, with the exception of sea buckthorn-based beverages, where the DPPH-EPR assay gave the lowest results. Rosehip-acerola beverages exhibited the highest AC values measured using all three methods, probably because of the combined action of vitamin C, polyphenols, and carotenoids. The AC of cranberry and sea buckthorn beverages were significantly lower compared to aronia and rosehip—acetrola products. This is consistent with earlier reports of other researchers, where aronia and acerola juices, as well as rosehip nectars, were characterized by higher AC than cranberry and sea buckthorn juices [54,55,56].

Generally, the traditional version showed a higher AC than the dietary one. Still, this tendency cannot be explained by the added sugar content, as it was shown, at least in the DPPH assay, that a concentration of sucrose of up to 45 g/kg of solution does not significantly increase the AC [57], while the addition of stevia in fruit juice can increase its AC [58]. Thus, this could be due to the stabilization of antioxidants in the presence of sugar.

Pasteurization did not affect the AC of most of the samples; a significant decrease was observed in only a few systems (Table 2). In one case, surprisingly, the increase in antioxidant capacity determined with the DPPH-EPR assay was observed, namely for the dietary version of the rosehip-acerola-based beverage. Still, for rosehip-acerola-based beverages, an increase in AC was observed in other assays as well, although it was not statistically significant. This could suggest that during pasteurization, some antioxidants present in the rosehip-acerola system transform into more active ones, such as those with a smaller molecular weight.

The pharmaceutically important compounds of herbs have not been assayed, although it should be borne in mind that a certain part of the polyphenols and the antioxidant capacity are derived from the herbal components.

The *F*-test values of physicochemical data are listed in Table S1 in the Supplementary Materials.

### 3.3. Consumer Acceptability Testing

The characteristics of the consumers invited to participate in the survey are shown in Table 3. A total of 60 people participated, 50% of which were men and 50% women. The panelists were aged from 18 to over 55; they were divided according to age into five groups of the same size, which also included 50% of the women and 50% of the men in each. The general recommendation is that this kind of testing should involve at least 100 people to ensure the representativeness of the potential consumers of the product [59,60]. However, according to Stone and Siedel [61], when testing is performed in laboratory conditions, as was the case in this study, the number of consumers could be smaller, and usually includes 30 to 50 people. The attitude towards body mass and health differed according to the consumer’s age and was the most positive in the 25- to 35-year-old age group and the oldest group—aged over 55. Only 8.4% of the participants were definitely not, or rather not interested in body mass and health care.

The overall acceptance (Table 4) was the result of the perception of all sensory features, taste, odour and appearance. All these attributes were significantly (*p* ≤ 0.05) correlated with overall acceptance. The correlation coefficients show that taste had the strongest impact on product acceptance (r = 0.85), followed by odour (r = 0.60) and colour (r = 0.54).

The beverages with the highest rated overall acceptance were those containing aronia juice; the average scores were 7.3 and 7.0 points for the sugar- and stevia-sweetened versions, respectively. The least liked were the beverages with sea buckthorn, gaining an average score of 3.2 and 3.0 for stevia and sugar sweetened versions, respectively. Overall acceptance of the remaining beverages was rated between 5.6 to 6.0 points and did not differ significantly. The low scores for sea buckthorn beverages was due to their specific sour taste and odour, perceived by most of the assessors as being unpleasant.

What is interesting is that there was no significant difference in the overall acceptance, or scores for the taste between sugar- and stevia-sweetened beverages. This may lead to the conclusion that steviol glycosides can be used to sweeten these types of products with a strong pungent and/or acidic taste, which masks the specific bitter, metallic aftertaste of this sweetener. Another explanation may be connected with the fairly widespread consumer awareness of the health benefits of consuming low-sugar products. A study carried out by Reis and co-workers [62] on the effect of the information about sugar reduction and the use of sweeteners on the consumer’s hedonic and sensory perception of wellbeing in the case of orange/pomegranate juice proved that information about the product’s nutritional characteristics had a positive effect on consumer perception. This was particularly visible when a natural sweetener such as stevia was used to replace sugar, while the consumers’ sensory and hedonic perception of the juice was not modified in the case of juices sweetened with an artificial sweetener, such as sucralose. The authors concluded that information about the nutritional characteristics of the products and reformulation strategies could have a positive effect on consumer perception, given that their sensory perception of the reformulated products does not differ largely from their regular counterparts.

Overall acceptance was reflected in the willingness to purchase the beverages (Table 4). Over half the consumers declared they would buy aronia beverages, while only 5% expressed such a wish with regard to stevia-sweetened sea buckthorn beverage, and no-one declared that they would buy a sugar-sweetened version. Thirty-seven percent of the tested consumers would buy rosehip-acerola beverages (with stevia), and 30% (with sugar) and 25% would buy cranberry beverages, and 22% for the traditional and diet versions, respectively.

There was no significant difference in the estimation of the overall acceptance of individual beverages between male and female consumers, although the average value for women was slightly but significantly higher (Table 5). There was no clear differentiation in the purchase declaration between the sexes. Several other studies indicate gender differences in dietary choices, which are attributed to the greater involvement of women in weight control, and to the fact that they pay more attention to healthy eating than men [63,64].

The other differentiating factor was the age of the evaluators (Table 6). The average rate of overall acceptance in the group of consumers in the 25–34 age group was the highest (6.1 points) and was significantly different from the average for the over-55 group. The results of the survey conducted in Poland [65] led to the conclusion that most of the respondents consuming functional foods were in the 26–35 and 46–55 age groups, which is consistent with our findings according to the younger group.

Care for proper body mass and health had no influence on the willingness to purchase beverages among people declaring high, moderate, rather low, or low concern of these aspects in their lives (Table 7). There was no such dependency, both in the group of people willing and not willing to make a purchase. The only beverage which consumers not taking care of their body and health would buy were the aronia ones, which were generally considered the tastiest; only a few consumers in the group considered the most health-oriented would buy sea buckthorn beverages, which were perceived as the least tasty. Similar results were reported by Menezes et al. [66] when they studied the acceptance values for açaí-based products. There was no statistically significant difference between consumers declaring a low and high general health interest; both groups appreciated the most-liked products, which in this case were the drinks with the lowest juice content. Similar results were observed with regard to purchase intentions. Another study on açaí beverages [34] shows that the flavour and taste experience were the most important in the overall score for the products, and not the consumers’ perceived health benefits; again, the most liked were the beverages with the lowest content of açaí juice. A review of the factors affecting food choice in relation to fruit and vegetable intake [30] also stresses that sensory perception is the most influential in determining eating behaviour. On the other hand, there is research indicating that product acceptance is evidently connected with information about the product’s composition, preservation methods, and potential health benefits. As shown in the example of orange juices, the consumers’ preferred samples were marked as being fresh juice over processed products, even when the same sample, being a mix of fresh and processed beverages, was served [67]. The acceptance of processed juices was higher when consumers were given information on the juice-processing characteristics, price, ingredients, and shelf-life. Consumer studies carried out using snacks with innovative functional ingredients, such as lupine and orange fibre [68], showed that people’s so-called “willingness to pay” for functional foods significantly varied with food values related to their origin, safety, naturalness, price, and so forth.

It is surprising that the developed beverages, which were accepted and at least moderately assessed according to their sensory quality by trained panelists, recruited from among employees of the Department of Fruit and Vegetable Product Technology of IBPRS, were not appreciated by external consumers, even those declaring an interest in their health. This can be explained by the fact that our employees were far more than moderately aware of the health benefits of these kinds of products, and were also more familiar with the various innovative fruit functional products which sometimes had an unconventional flavour.

## 4. Conclusions

The developed functional fruit-herbal beverages, containing a high percentage of fruit juices and supplemented with plant extracts of known health-promoting benefits, were a valuable source of polyphenols, anthocyanins, carotenoids, and vitamin C, depending on the type of used fruit. All the beverages exhibited high antioxidant potential. The energy value of the diet version of the beverages, sweetened with stevia glycosides, was at least 30% lower than the traditional version with added sugar. The consumer tests carried out on 60 adults showed that aronia beverages were the most accepted ones and could find buyers when introduced to the market. The other beverages were poorly or not accepted by the majority of the persons testing them, despite information on the pro-health effects of the products. The beverages were rated slightly higher by women than men, and by people of ages between 25–34. There were no meaningful differences between the gender and age of consumers, as was the willingness to buy them.

The study has some limitations resulting from the smaller size of the consumer group, consisting of 60 volunteers, which was the effect of the limited capacity of the sensory laboratory. Additionally, the consumers were recruited among the citizens of a big city, Warsaw, and its suburbs, so the conclusions cannot extend to the entire Polish population.

The general conclusion is that, despite growing consumer awareness of the pro-health properties of fruit products, the main motive for purchasing them is still sensory acceptance, even in the case of functional products with declared health benefits.

## Figures and Tables

**Table 1:** Composition of fruit-herbal functional beverages.

Kind of Beverage	Juice (g/kg)	Version	Sweetener (g/kg)		Herbal and Plant Extracts
			Sugar	Steviol Glycoside	
Aronia	800	T	35	-	cistus, green tea, nettle, artichoke
		D	-	0.175	
Rosehip and acerola	600:200	T	30	-	Siberian ginseng, ginger, purple coneflower, aloe
		D	-	0.15	
Cranberry	800	T	15	-	horsetail, lingonberry, silver birch, chamomile
		D	-	0.75	
Sea buckthorn	800	T	50	-	horsetail, chamomile, nettle
		D	-	0.25	

T-traditional (with sugar); D-dietary (with steviol glycosides).

**Table 2:** Physicochemical characteristic of fruit-herbal functional beverages.

Type of Beverage		Vitamin C (mg/L)		Carotenoids (mg/L)		Polyphenols (mg GA/L)		Anthocyanins (mg/L)		Antioxidant Capacity (µM Trolox/g)					
										ABTS		DPPH		ORAC	
		O	P	O	P	O	P	O	P	O	P	O	P	O	P
Aronia	T	-	-	-	-	696 ± 9 a	691 ± 5 a	220 ± 4 a	137 ± 3 b	48.0 ± 8.0 a	47.3 ± 0.9 a	79.0 ± 2.0 a	66.3 ± 0.6 b	79.5 ± 1.0a	73.0 ± 8.0 ab
	D	-	-	-	-	677 ± 20 a	668 ± 19 a	145 ± 3 b	116 ± 3 c	54.1 ± 0.2 b	57.6 ± 1.9 c	71.4 ± 3.4 c	71.4 ± 0.4 c	68.3 ± 5.3b	66.6 ± 0.6 b
Rosehip−acerola	T	3597 ± 4 a	3439 ± 1 b	82 ± 3 a	78 ± 2 a	803 ± 9 a	807 ± 13 a	-	-	58.8 ± 2.7 a	63.2 ± 1.9 b	89.0 ± 7.0 a	75.7 ± 2.6 b	101.1 ± 9.3ab	104.9 ± 2.5 a
	D	3707 ± 2 c	3540 ± 6 d	80 ± 1 a	75 ± 3 a	817 ± 7 a	812 ± 9 a	-	-	64.1 ± 1.1 b	65.0 ± 4.2 b	70.0 ± 5.0 c	94.0 ± 8.0 a	94.0 ± 7.0bc	91.0 ± 1.9 c
Cranberry	T	-	-	-	-	245 ± 9 a	230 ± 9 ab	43.6 ± 0.1 a	22.4 ± 0.2 b	16.3 ± 0.3 a	15.5 ± 2.1 abc	20.0 ± 1.1 a	17.9 ± 1.4 bc	45.9 ± 9.0ab	41.3 ± 1.4 a
	D	-	-	-	-	250 ± 13 a	213 ± 6 b	39.7 ± 0.6 c	24.4 ± 0.2 d	14.0 ± 0.2 b	15.5 ± 0.3 c	15.1 ± 3.2 b	19.1 ± 1.8 ac	39.1 ± 0.9b	31.0 ± 2.2 c
Sea buckthorn	T	1206 ± 4 a	1194 ± 9 a	65 ± 3 ab	60 ± 3 a	339 ± 6 a	341 ± 5 a	-	-	22.0 ± 1.1 a	21.3 ± 0.3 a	18.9 ± 0.8 a	18.9 ± 2.5 ab	45.9 ± 1.4ab	45.3 ± 1.6 ab
	D	1111 ± 7 b	1094 ± 8 b	68 ± 2 b	59 ± 1 a	341 ± 10 a	331 ± 8 a	-	-	20.2 ± 0.7 b	19.5 ± 0.8 b	18.8 ± 1.0 a	17.6 ± 0.7 b	44.0 ± 3.0a	46.8 ± 0.7 b

O—fresh; P—pasteurized; T-traditional (with sugar); D-dietary (with steviol glycosides). The same letters indicate there is no significant difference between the mean values of the parameter among the beverages prepared from the same fruit juice.

**Table 3:** Characteristics of the consumer group.

Age	Gender (%)		Care for Body Mass and Health (%)			
	Male	Female	Yes	Rather Yes	Rather No	No
18–24	50	50	41.7	50	8.3	-
25–34	50	50	50	50	-	-
35–44	50	50	16.7	75	8,3	-
45–54	50	50	25	50	16.7	8.3
>55	50	50	58.3	41.7	-	-
all	50	50	38.3	53.3	6.7	1.7

**Table 4:** Consumer acceptance of fruit-herbal beverages and willingness to purchase.

Kind of Beverage		Taste (0–10 Point) F_0.05_(7472) = 50.950	Odour (0–10 Point) F_0.05_(7472) = 26.956	Colour (0–10 Point) F_0.05_(7472) = 39.706	Overall Acceptance (0–10 Point) F_0.05_(7472) = 45.965	Willingness to Purchase (%)
Aronia	T	7.3 ± 1.7 c	6.2 ± 2.1 b	8.3 ± 1.4 c	7.3 ± 1.4 c	53.3
	D	6.6 ± 1.8 c	5.7 ± 2.1 b	8.0 ± 1.8 c	7.0 ± 1.9 c	56.7
Rosehip−acerola	T	5.5 ± 2.0 b	5.9 ± 2.0 b	6.2 ± 1.8 b	5.6 ± 1.9 b	30.0
	D	5.6 ± 2.2 b	5.8 ± 2.0 b	5.7 ± 1.7 ab	5.7 ± 2.2 b	36.7
Cranberry	T	4.9 ± 1.9 b	7.3 ± 1.7 d	8.0 ± 1.4 c	5.9 ± 1.8 b	26.7
	D	5.1 ± 1.9 b	7.0 ± 1.9 cd	8.0 ± 1.5 c	6.0 ± 1.6 b	23.3
Sea buckthorn	T	2.4 ± 1.7 a	3.7 ± 2.1 a	5.1 ± 2.2 a	3.0 ± 1.7 a	0
	D	2.7 ± 1.9 a	3.8 ± 1.9 a	5.1 ± 2.0 a	3.2 ± 1.8 a	5.0

T-traditional (with sugar); D-dietary (with steviol glycosides). The same letters indicate no significant difference between mean values of the parameter in the column.

**Table 5:** Consumer acceptance and willingness to purchase fruit-herbal beverages depending on gender.

Type of Beverage		Overall Acceptance (0–10 Point)				Willingness to Purchase (%)	
		Male		Female		Male	Female
		Average	F_0.05_(1,58)	Average	F_0.05_(1,58)		
Aronia	T	7.1 ± 1.7 a	1.540	7.5 ± 1.1 a	0.010	60.0	46.7
	D	6.5 ± 2.1 a		7.5 ± 1.5 a		50.0	63.3
Rosehip−acerola	T	5.3 ± 1.9 a	0.256	6.0 ± 2.0 a	0.422	26.7	33.3
	D	5.0 ± 2.2 a		6.3 ± 2.0 a		33.3	40.0
Cranberry	T	5.8 ± 1.7 a	0.145	6.0 ± 1.9 a	0.006	30.0	23.3
	D	5.9 ± 1.7 a		6.0 ± 1.6 a		30.0	16.7
Sea buckthorn	T	3.0 ± 1.7 a	0.089	3.0 ± 1.8 a	0.518	0	0
	D	3.1 ± 1.7 a		3.3 ± 1.8 a		6.7	3.3
Average		5.2 ± 2.3 a	-	5.7 ± 2.3 b	-	29.6	28.3

T-traditional (with sugar); D-dietary (with steviol glycosides); the same letters indicate no significant difference between the mean values of the parameter among one type of beverage and between average values.

**Table 6:** Consumer acceptance and willingness to purchase fruit-herbal beverages depending on age.

Type of Beverage	Age	Average	Aronia		Rosehip−Acerola		Cranberry		Sea Buckthorn	
			T	D	T	D	T	D	T	D
	**18–24**	5.5 ± 2.5 ab	8.3 ± 0.7 b	7.5 ± 1.4 a	5.4 ± 1.8 a	5.3 ± 2.3 a	6.3 ± 2.3 a	6.2 ± 1.5 a	2.6 ± 1.5 a	2.8 ± 1.9 a
**Overall acceptance** **(0–10 point)**	**25–34**	6.1 ± 2.2 b	7.0 ± 1.6 ab	7.7 ± 2.1 a	6.3 ± 1.6 a	6.9 ± 2.0 a	6.3 ± 1.9 ba	6.7 ± 1.7 a	3.6 ± 1.8 a	4.2 ± 1.8 a
	**35–44**	5.2 ± 2.6 ab	7.2 ± 1.6 ab	6.7 ± 2.2 a	5.5 ± 2.8 a	5.5 ± 2.7 a	5.6 ± 1.8 a	5.8 ± 1.9 a	2.8 ± 2.1 a	2.8 ± 1.6 a
	**45–54**	5.4 ± 2.1 ab	7.3 ± 1.1 ab	6.8 ± 1.0 a	6.1 ± 1.4 a	6.1 ± 1.6 a	5.3 ± 1.5 a	5.5 ± 1.5 a	3.0 ± 2.0 a	3.3 ± 2.0 a
	**≥55**	5.0 ± 2.0 a	6.7 ± 1.6 a	6.3 ± 2.2 a	4.8 ± 1.6 a	4.5 ± 1.7 a	5.8 ± 1.4 a	5.8 ± 5.8 a	2.9 ± 1.2 a	3.1 ± 1.4 a
**F_0.05_(4,55)**		3.124	2.612	1.155	1.256	2.290	0.831	0.979	0.530	1.347
	**18–24**	32.3	58.3	83.3	33.3	50.0	16.7	8.3	0.0	8.3
**Willingness** **to purchase** **(%)**	**25–34**	27.1	25.0	58.3	16.7	50.0	16.7	33.3	0.0	16.7
	**35–44**	32.3	75.0	41.7	33.3	25.0	50.0	33.3	0.0	0.0
	**45–54**	27.1	58.3	58.3	41.7	41.7	8.3	8.3	0.0	0.0
	**≥55**	26.0	50.0	41.7	25.0	16.7	41.7	33.3	0.0	0.0

T-traditional (with sugar); D-dietary (with steviol glycosides); the same letters indicate no significant difference between the mean values of the parameter among one type of beverage.

**Table 7:** Consumer willingness to purchase beverages depending on care for proper body weight and health.

		Willingness to Purchase (%)															
		Yes								No							
	Type of Beverage	Aronia		Rosehip−Acerola		Cranberry		Sea Buckthorn		Aronia		Rosehip−Acerola		Cranberry		Sea Buckthorn	
Attitude Toward Proper Body Mass and Health		T	D	T	D	T	D	T	D	T	D	T	D	T	D	T	D
% care for proper body mass and health		25.0	38.2	38.9	40.9	37.5	42.9	0	66.7	53.6	38.5	38.1	36.8	38.6	37	36.8	36.8
% rather care for proper body mass and health		62.5	55.9	50.0	50	56.3	57.1	0	33.3	42.9	50	54.8	55.3	52.3	52.2	54.4	54.4
% rather do not care for proper body mass and health		9.4	2.9	11.1	9.1	6.3	0	0	0	3.6	11.5	4.8	2.6	6.8	8.7	7.0	7.0
% do not care for proper body mass and health		3.1	2.9	0	0	0	0	0	0			2.4	36.8	2.3	2.2	1.8	1.8
**Chi^2(Chi-square)**		2.1575		0.5050		0.9296				1.9686		0.0243		0.1197		0.0299	
***df***		3		2		2				2		3		3		3	
***p***		0.540		0.975		0.628				0.374		0999		0.989		0.999	

T-traditional (with sugar); D-dietary (with steviol glycosides).

## Supplementary Materials

The following are available online at https://www.mdpi.com/2304-8158/9/12/1819/s1, Table S1: *F*-test values of analysis of physicochemical characteristic of fruit-herbal functional beverages presented in Table 2.
