# Supplementary material for: The Development and Consumer Acceptance of Functional Fruit-Herbal Beverages
Source: Foods. 2020 Dec 8;9(12):1819. doi: 10.3390/foods9121819 (PMC7762522; doi:10.3390/foods9121819)
Supplement: Supplementary file 1 [file foods-09-01819-s001.pdf]

Table S1: F-test values of analysis of physicochemical characteristic of fruit-herbal functional beverages presented in Table 2

Type of beverage		Vitamin C	Carotenoids	Polyphenols	Anthocyanins	Antioxidant capacity		
						ABTS	DPPH	ORAC
		O P	O P	O P	O P	O P	O P	O P
Aronia	T D	-	-	$F_{0.05}(1,8) = 0.04$	$F_{0.05}(1,4) = 145.79$	$F_{0.05}(1,8) = 20.62$	$F_{0.05}(1,8) = 31.65$	$F_{0.05}(1,8) = 5.62$
Rosehip - acerola	T D	$F_{0.05}(1,4) = 17.00$	$F_{0.05}(1,4) = 0.52$	$F_{0.05}(1,8) = 0.55$	-	$F_{0.05}(1,8) = 5.34$	$F_{0.05}(1,8) = 31.56$	$F_{0.05}(1,8) = 6.68$
Cranberry	T D	-	-	$F_{0.05}(1,8) = 5.41$	$F_{0.05}(1,4) = 70.74$	$F_{0.05}(1,8) = 8.24$	$F_{0.05}(1,8) = 8.65$	$F_{0.05}(1,8) = 6.16$
Sea buckthorn	T D	$F_{0.05}(1,4) = 37.00$	$F_{0.05}(1,4) = 9.31$	$F_{0.05}(1,8) = 2.08$	-	$F_{0.05}(1,8) = 6.15$	$F_{0.05}(1,8) = 6.17$	$F_{0.05}(1,8) = 9.00$
